# Depressive symptoms as a barrier to engagement in physical activity in older adults with and without Alzheimer’s disease

**DOI:** 10.1371/journal.pone.0208581

**Published:** 2018-12-07

**Authors:** Amber S. Watts, Moyra E. Mortby, Jeffrey M. Burns

**Affiliations:** 1 University of Kansas Alzheimer’s Disease Center, Fairway, KS, United States of America; 2 School of Psychology, University of New South Wales and Neuroscience Research Australia (NeuRA), Randwick, Sydney, NSW, Australia; Nathan S Kline Institute, UNITED STATES

## Abstract

**Objectives:**

Physical activity shows promise for reduced risk of Alzheimer’s disease (AD) and protection against cognitive decline among individuals with and without AD. Older adults face many barriers to adoption of physically active lifestyles and people with AD face even further challenges. Physical activity is a promising non-pharmacological approach to improve depressive symptoms, but little is known about the impact of depressive symptoms as a potential barrier to engagement in physical activity. The present study aimed to investigate depressive symptoms as a potential barrier for participation in physical activity across a range of dementia severity.

**Method:**

We used longitudinal structural equation modelling to investigate the bi-directional relationship between depressive symptoms and physical activity in 594 older adults with and without AD over a 2 year longitudinal follow up. Participants ranged from no cognitive impairment to moderately severe AD.

**Results:**

We found that depressive symptoms predicted reduced engagement in subsequent physical activity, but physical activity did not predict subsequent reductions in depressive symptoms.

**Conclusion:**

We conclude that depressive symptoms may be an important barrier to engagement in physical activity that may be addressed in clinical practice and intervention research.

## Introduction

Older adults spend 65–80% of waking time in sedentary activities [1,2], and individuals with Alzheimer’s disease (AD) are even less active [3,4]. Physical activity shows promise for reduced risk of AD and protection against cognitive decline among individuals with and without AD [5,6]. However, older adults face many barriers to adoption of physically active lifestyles. In one study, 87% of older adults reported one or more barriers to exercise [7]. In addition to the barriers to physical activity faced by most people, (e.g., perceived effort, discomfort of sweating, muscle soreness), older adults face unique barriers such as chronic health problems, fear of falling, inadequate environmental support, and lack of self-efficacy about exercise [7,8].

People with AD likely face even further challenges to engaging in physical activity. Aside from a few small qualitative studies [9–11], there is little research addressing the barriers to physical activity specific to individuals with dementia [7]. The barriers reported in qualitative studies fall into several categories including physical, social, emotional, environmental, and cognitive. The physical barriers are likely similar to those reported in older adults without dementia such as decreased energy and impaired body function [11]. Malthouse and Fox [9] reported that people with AD generally had positive attitudes about the benefits of physical activity, however few of them were willing to push themselves hard enough to sweat and did not like the feeling of being forced to exercise.

However, barriers due to cognitive disability and socio-emotional factors are more unique to persons with AD. For example, a hallmark cognitive symptom of AD is disorientation to place. This makes it difficult and unsafe for individuals with AD to walk for exercise without a companion or in an unfamiliar environment. A key social-emotional barrier to physical activity is the need to have a caregiver to arrange for transportation, accompaniment, and assurance of safety. AD patients reported feeling a loss of freedom and safety in their inability to exercise unsupervised and loss of identity in their inability to continue with activities they previously enjoyed [11]. The development of “dementia-friendly” communities would be beneficial for allowing people to regain some sense of independence.

Individuals with AD have been found to have a reduced ability to accurately perceive bodily states that impact effort during physically demanding activity [12]. This may result in over- or under- exertion during exercise leading to unsafe conditions or insufficient effort to achieve benefits of exercise. There is also evidence that cognitive and motor declines in dementia may occur in parallel due to underlying brain changes [13]. Some possible mechanistic explanations of this process include changes in brain and spinal cord areas that regulate motor movement, and vulnerability of muscle to systemic dysregulation of catabolic, inflammatory, immune, endocrine, and metabolic processes. Loss of lean mass, muscle strength, and motor performance are common in older adults and a prominent features of frailty, sarcopenia, and metabolic syndrome, all of which are associated with risk of AD [14].

Limitations of attention and memory in people with AD may require additional prompts for carrying out multistep daily tasks [15]. With increasing disease severity, individuals with AD become less likely to initiate activities of daily living, social engagement, or other daily activities, and become increasingly dependent on support of caregivers to carry out these tasks [16]. Reduced initiation in activity engagement may be related to depressive symptoms, cognitive impairment, or other disease-related neurological changes [17,18]. Rates of depression are higher among individuals with AD compared to those with normal cognitive status, though the direction of the causal relationship between dementia and depression remains unclear [19,20].

Physical activity and exercise are a promising non-pharmacological approach to improve depressive symptoms in older adults with or without dementia, without the side effects or drug interactions commonly found with pharmacological therapy in this population [21]. A great deal of research has demonstrated benefits of physical activity and exercise for improving depressive symptoms in non-cognitively-impaired older adults and in individuals with AD and other forms of dementia [22,23].

Despite the extensive research into the protective nature of exercise for dementia, and the promise of physical activity for reducing depressive symptoms, to date, little is known about the impact of depressive symptoms on engagement in physical activity. It is plausible, that given the prevalence of depressive symptoms among individuals with dementia, these symptoms may present additional challenges to engaging in physical activity. For example, the presence of symptoms such as dysphoric mood, apathy, and psychomotor retardation may further prevent older adults with or without AD from engaging in physical activity. This is of particular clinical relevance as the presence of such depressive symptoms may limit the efficacy of physical activity as a non-pharmacological intervention.

This study had two aims. Aim 1 was to evaluate the effect of engagement in physical activity on subsequent depressive symptoms, thereby confirming previous findings that physical activity is associated with reduced depressive symptoms. Aim 2 was to examine the reverse hypothesis that depressive symptoms act as a potential barrier to subsequent engagement in physical activity.

## Methods

### Participants

All study procedures approved by the University of Kansas Medical Center Human Subjects Committee #11132. Written consent was obtained for all participants and/or their legally authorized representative. All participants were enrolled in a Midwestern Alzheimer’s Disease Center Clinical Cohort and received standard clinical and cognitive evaluations annually. Detailed information about evaluation procedures and data collection have been published previously [24]. Briefly, experienced study clinicians trained in dementia assessment conducted a standard clinical evaluation that includes a Clinical Dementia Rating (CDR) [25] and a trained psychometrician administered a comprehensive cognitive testing battery. The clinical and psychometric test results were reviewed and discussed at a weekly consensus conference that included clinicians, a neuropsychologist, and raters to determine a final consensus diagnosis. AD diagnosis was made using National Institute of Neurological and Communicative Disorders and Stroke and the Alzheimer’s Disease and Related Disorders Association criteria (NINCDS-ADRDA) [26]. Dementia severity was determined using the CDR. Because they represented a very small proportion of the sample, we excluded patients with dementia due to other causes including vascular dementia, Lewy Body dementia, and frontotemporal dementia. To assign an etiology for the disease, we followed the National Institute on Aging-Alzheimer’s Association (NIA-AA) workgroup diagnostic guidelines for Alzheimer’s disease which include the category “mild cognitive impairment due to Alzheimer’s disease” to identify individuals with symptoms consistent with AD pathophysiology, but not yet meeting criteria for dementia [27]. Participants with active ischemic heart disease or uncontrolled insulin dependent diabetes mellitus were excluded.

The present study includes 594 individuals categorized as having either no impairment (CDR = 0; n = 345), mild cognitive impairment due to AD (CDR = 0.5; n = 154), and AD (CDR = 1 or greater; n = 95). Data are reported from three waves of annual assessment. Baseline participant characteristics are shown in Table 1. All characteristics described in Table 1 differed statistically by CDR status and p < .001.

**Table 1:** Baseline participant characteristics.

	Total Sample N = 594 M (SD)	CDR = 0 N = 345 M (SD)	CDR = 0.5 N = 154 M (SD)	CDR = 1+ N = 95 M (SD)
Age	72.70 (7.39)	72.35 (6.49)	71.89 (8.11)	75.29 (8.64)
Education (years)	16.13 (2.96)	16.53 (2.84)	15.92 (3.07)	15.01 (2.90)
Physical Activity (RAPA score)	3.86 (1.90)	3.98 (1.84)	4.06 (1.95)	3.08 (1.90)
Geriatric Depression Scale Score	1.39 (2.05)	0.81 (1.29)	2.59 (2.29)	1.57 (1.69)
	**N (%)**	**N (%)**	**N (%)**	**N (%)**
Female	353 (59.4)	234 (67.8)	67 (43.5)	52 (54.7)
Neuropsychiatric Medication Use	203 (34.2)	85 (24.6)	70 (45.5)	48 (50.5)

Notes: RAPA scores range from 0 (rarely or never do physical activities) to 7 (I do 20 minutes or more a day of vigorous physical activities, 3 or more days a week). A score of 4 corresponds to the statement, “I do moderate physical activity every week, but less than 30 minutes a day or 5 days a week”. Responses to the Geriatric Depression Scale in our sample ranged from 0 to 13. Classes of medications included benzodiazepines, tricyclics, selective serotonin reuptake inhibitors (SSRIs), atypical antipsychotics, serotonin-norepinephrine reuptake inhibitors (SNRIs), and bupropion.

All study procedures were conducted in accordance with the Helsinki Declaration and approved by the institutional Human Subjects Committee. Participants and their legally authorized representatives completed an informed consent process before participating in the study. The potential participant’s understanding of the research was established by obtaining verbal responses that paraphrase the material in a way that indicates understanding. These procedures have been approved by our institutional review board.

### Measures

The Geriatric Depression Scale (GDS) was administered by a trained coordinator to an informant knowledgeable about the participant’s behavior. The GDS is a widely used measure of depressive symptoms in older adults. We used the 15 item version to evaluate depressive symptoms [28]

To assess physical activity engagement, we used the Rapid Assessment of Physical Activity (RAPA), which was designed to assess physical activity in older adults [29]. It defines light, moderate, and vigorous activities for participants according to the degree of perceived heart and respiratory rates achieved during activity for pleasure, work, or transportation. It classifies respondents according to their highest level of activity over a typical week according to the intensity level, duration, and frequency of activity. There is a lack of research validating physical activity assessment tools for use in people with dementia [30]. Thus, we relied on a common practice which is to have questionnaires completed by both patients and their caregivers with guidance from the study personnel.

Covariates included self-reported age, sex, and years of education, clinically assessed body mass index (BMI), and use of antidepressant and antianxiety medications. Classes of medications included benzodiazepines, tricyclics, selective serotonin reuptake inhibitors (SSRIs), atypical antipsychotics, serotonin-norepinephrine reuptake inhibitors (SNRIs), and bupropion.

### Statistical analysis

We report findings from three waves of assessment, each one year apart. We examined longitudinal relationships between depressive symptoms and physical activity to help establish causal direction. We used confirmatory factor analysis (CFA) to summarize the score for the GDS using a single factor. CFA is advantageous for providing improved measurement accuracy by aggregating common variance across multiple items and attenuating error idiosyncratic to individual items. Using a total score assumes that each item is equally valuable in contributing to the construct of depression, whereas CFA does not make that assumption. To evaluate model fit we used Root Mean Squared Error of Approximation (RMSEA), a measure of the discrepancy between predicted and observed model values. Values closer to 0 indicate better fit (preferred values <0.09) We also report comparative fit index (CFI) which estimates the relative fit of a model compared to an alternative model (CFI >0.90 indicates good fit). We used longitudinal structural equation modeling to evaluate the association between depressive symptoms (GDS) and physical activity (RAPA) adjusting for age, sex, education, BMI, CDR, and use of antidepressant and antianxiety medications. To help establish causal direction of influence, we tested the effect of physical activity on subsequent depressive symptoms and tested the reverse, the effect of depressive symptoms on subsequent physical activity. Because we are using structural equation modeling to estimate all the pathways simultaneously, we do not have the same inflation of type 1 error associated with multiple testing. We used Full Information Maximum Likelihood to statistically represent missing data.

## Results

Baseline demographic characteristics are given in Table 1. A one factor model provided good fit to the items on the GDS (χ^2^ [df] = 112.737 [90], RMSEA = 0.021, CFI = 0.984). All items loaded on the single factor with estimates of .56 or higher. At baseline, we had data for 594 participants. At the second and third waves of data collection, we had 423 and 311 observations, respectively.

**Fig 1:**
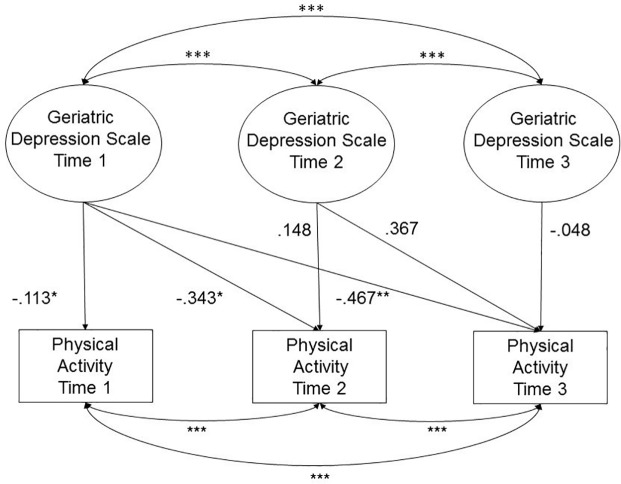
Geriatric depression scale predicts physical activity adjusting for age, sex, education, BMI, CDR, neuropsychiatric medications. Presented in standardized estimates. * p < .05, ** p < .01, *** p < .001.

To evaluate the direction of influence between physical activity and depressive symptoms, we tested the relationships between each at three waves of assessment adjusting for age, sex, education, CDR, BMI, and use of neuropsychiatric medications. A summary of the neuropsychiatric medications used in the sample can be found in S1 Table. Results of both models are reported in Table 2. The first three columns represent the effects of physical activity and covariates on depressive symptoms across three waves (Model 1). Physical activity did not predict depressive symptoms at any of the three time points. The second three columns represent the effects of depressive symptoms on physical activity across the three waves (Model 2). Higher levels of baseline depressive symptoms on the GDS predicted lower levels of physical activity at all three waves (Wave 1 β = -.188, p < .05, Wave 2 β = -.165, p < .05, β = -.213, p < .01). See Fig 1 for an illustration of Model 2 in which the GDS at each time point predicts concurrent and subsequent physical activity scores. Higher dementia severity (CDR) was consistently associated with a higher number of depressive symptoms. Higher BMI was consistently associated with lower rates of physical activity.

**Table 2:** Results of structural equation models of physical activity as a predictor of depressive symptoms on the GDS and depressive symptoms on the GDS as a predictor of physical activity across three waves (standardized estimates).

	Depressive symptoms Time 1		Depressive symptoms Time 2		Depressive symptoms Time 3		Physical activity Time 1		Physical activity Time 2		Physical activity Time 3	
Physical activity time 1	-.128		-.018		-.015		—		—		—	
Physical activity time 2	—		-.043		-.107		—		—		—	
Physical activity time 3	—		—		.023		—		—		—	
Depressive symptoms time 1	—		—		—		-.188	*	-.165	*	-.213	**
Depressive symptoms time 2	—		—		—		—		-.059		.055	
Depressive symptoms time 3	—		—		—		—		—		.010	
Age	-.053		-.037		-.031		-.127		-.165	**	-.193	***
Sex	.019		.001		-.072		-.069		-.115		-.078	
Education (years)	-.107		-.049		-.005		.106		.017		.137	*
CDR	.218	**	.287	***	.192	*	-.080		-.046		-.143	*
BMI	-.050		-.038		.080		-.187	**	-.160	**	-.203	***
Neuropsychiatric Medication	.124		.139		.166	*	-.071		-.100		-.150	**

* p < .05

** p < .01

*** p < .001; Note: Classes of medications included benzodiazepines, tricyclics, selective serotonin reuptake inhibitors (SSRIs), atypical antipsychotics, serotonin-norepinephrine reuptake inhibitors (SNRIs), and bupropion.

## Discussion

To the best of our knowledge, this is the first study to investigate the directional relationship between depressive symptoms and participation in physical activity across a range of dementia severity using a longitudinal approach. In contrast to commonly reported benefits of physical activity for improvement of depressive symptoms, we found that in our sample, depressive symptoms predicted reduced engagement in later physical activity participation over a two year follow up. Thus, we conclude that depressive symptoms may be an important barrier to engagement in physical activity that may be addressed in clinical practice and intervention research. Identifying and treating depressive symptoms may serve as a way to reduce barriers to physical activity which has been shown to have numerous benefits for health and cognitive function in older adults with and without cognitive impairment.

Although many studies have investigated barriers to physical activity among older adults, few studies have directly addressed depressive symptoms as a barrier and despite the high rate of comorbidity with dementia, we found no studies that considered this potential barrier in individuals with AD. The EPESE study [31] provided evidence that depressive symptoms predicted subsequent declines in physical performance including poorer balance, slower walking speed, and slower speed when rising from a chair. A possible explanation offered was a lower rate of walking, gardening, and vigorous exercise activities among individuals with a greater number of depressive symptoms. A prospective, population-based study of Norwegian adults reported that mood and exercise were correlated over a three year period, but did not find evidence for a consistent directional relationship between them [32]. One study, using a focus group methodology, reported negative affect as a barrier to engagement in exercise among older adults [33]. Our study adds to the sparse existing literature by considering the longitudinal and potentially bi-directional relationship between depressive symptoms and physical activity across individuals with a range of cognitive status.

There are several possible mechanisms that could reasonably explain how depressive symptoms may act as a barrier to physical activity, including physiological and psychological causes. Compared to younger adults, depression in older adults is characterized by fewer affective symptoms and more somatic symptoms.[34] Somatic symptoms of depression such as fatigue, change in body weight, slowed motor response, or poor sleep may contribute to reduced physical activity. Depression is associated with increased perception of pain [35], another known barrier to engagement in physical activity. Pharmacological treatments for depression may also result indirectly in reductions in physical activity. Common side effects of anti-depressant medications include weight gain, orthostatic hypotension, and other cardiovascular side effects that may alter the body’s response to physical activity [36,37].

Psychological mechanisms by which depressive symptoms prevent physical activity include apathy, anhedonia, and reduced self-efficacy. Apathy, characterized by reduced initiation and persistence, has been associated with reductions in self-care, functional impairment in activities of daily living, [18] and poorer cognitive function, all of which are likely barriers to activity. Anhedonia, a reduced ability to experience pleasure, may also reduce engagement in activities that were previously found to be pleasurable. Individuals with anhedonia tend to expect that they will not enjoy activities, thus becoming less likely to engage in them. Self-efficacy, an individual’s belief in their ability to successfully perform a specific behavior, has been identified as an important barrier to exercise in older adults.

Our findings have important implications for clinical practice and intervention research. To capitalize on the benefits of physical activity for health, depressive symptoms, and cognitive function, we must first remove barriers to engagement in physical activity that are inherent in our population, including the barriers created by the very symptoms we are hoping to treat. Before embarking on a program of physical activity, existing depressive symptoms need to be identified and treated. There are a number of approaches for the treatment of depressive symptoms in people with dementia, including both pharmacological and non-pharmacological approaches [38]. These include multi-sensory stimulation, Behavioral Therapy-Pleasant Events [38], and the combination of Shiatsu and physical activity [39].

To encourage adoption of physical activity routines among individuals with depressive symptoms, we need to educate people with depressive symptoms about the possible benefits for improvement of mood, physical, and cognitive symptoms. Because dysphoric mood is accompanied by a bias toward negative information and expectation that activities will not be pleasurable, physical activity routines should capitalize on experiences pleasurable to individuals. This might include activities that are socially engaging, interactive with nature or pets, or accompanied by music [40,41]. Physical activities must be appropriate to the physical and cognitive ability level of individuals, and ensure safety. Walking is the most common physical activity among older adults [3,42] and may be safe for individuals with AD in the right physical setting (e.g., safe, navigable) [43] or with a companion. Caregivers and other social supports play a critical role in the initiation and maintenance of any lifestyle routine.

### Limitations and future directions

A limitation of the study is the reliance on a self- and caregiver- reported measure of physical activity. Objective measures of physical activity such as accelerometry would improve accuracy of estimates. Our sample is largely Caucasian and well educated, reducing our ability to generalize our findings to broader samples of older adults. As is typical in clinical samples, we have a larger number of individuals without impairment compared to the number with impairment. This imbalance of sample size could potentially impact the results of our statistical analysis, though the analytic approach we used is generally robust to unbalanced sample sizes. Finally, our sample includes participants on the mild end of the dementia spectrum, thus we cannot draw conclusions about more severe stages of dementia.

Our understanding of how to increase physical activity engagement in older adults with and without AD would benefit from future research on ways to reduce depressive symptoms as a barrier to physical activity. To effectively increase the levels of physical activity among older people with and without AD, further research is needed to determine how to motivate persons within this population to exercise regularly despite the complexity of chronic health and mental health conditions [44]. Future research should consider individual symptoms specifically related to reduced physical activity and how to best treat them with medications or psychosocial interventions [38].

## Supporting information

S1 Table Summary of neuropsychiatric symptoms used in our sample. (DOCX)
