# Supplementary material for: Depressive symptoms as a barrier to engagement in physical activity in older adults with and without Alzheimer’s disease
Source: PLoS One. 2018 Dec 7;13(12):e0208581. doi: 10.1371/journal.pone.0208581 (PMC6286143; doi:10.1371/journal.pone.0208581)
Supplement: S1 Table — (DOCX) [file pone.0208581.s001.docx]

**Supporting Information**

| **Class of Drug** | **Number** |
| --- | --- |
| Benzodiazepines | 36 |
| SSRI | 110 |
| SNRI | 15 |
| Bupropion | 39 |
| Atypical antipsychotic | 7 |
| Tricyclic | 8 |
|  | 215 |
